# RGD-functionalised melanin nanoparticles for intraoperative photoacoustic imaging-guided breast cancer surgery

**DOI:** 10.1007/s00259-021-05545-3

**Published:** 2021-09-10

**Authors:** Jing-Jing Liu, Zun Wang, Li-Ming Nie, Yuan-Yuan Zhu, Ge Li, Lin-Ling Lin, Min Chen, Guo-Jun Zhang

**Affiliations:** 1 Cancer Center & Department of Breast and Thyroid Surgery, Xiang’an Hospital of Xiamen University, School of Medicine, Xiamen University, 2000 Xiang’an Road East, Xiamen, 361101 Fujian China; 2 Xiamen Key Laboratory for Endocrine-Related Cancer Precision Medicine, Xiang’an Hospital of Xiamen University, Xiamen, 361101 Fujian China; 3 ChangJiang Scholar’s Laboratory, Shantou University Medical College, Shantou, 515041 Guangdong China; 4 Present Address: Department of Breast and Thyroid Surgery, Shenzhen Baoan Women’s and Children’s Hospital, Jinan University, Shenzhen, 518133 Guangdong China; 5 State Key Laboratory of Molecular Vaccinology and Molecular Diagnosis & Center for Molecular Imaging and Translational Medicine, School of Public Health, Xiamen University, Xiamen, 361102 Fujian China; 6 Present Address: Department of Radiology and Optical Imaging Laboratory, Guangdong Provincial People’s Hospital, Guangdong Academy of Medical Sciences, Guangzhou, 510080 Guangdong China; 7 Cancer Research Center, School of Medicine, Xiamen University, Xiamen, 361101 Fujian China; 8 Clinical Central Research Core, Xiang’an Hospital of Xiamen University, 2000 Xiang’an Road East, Xiamen, 361101 Fujian China

**Keywords:** Melanin, Bioimaging, Photoacoustic imaging, Breast cancer, Tumour detection, Imaging-guided surgery

## Abstract

**Purpose:**

Obtaining tumour-free margins is critical for avoiding re-excision and reducing local recurrence following breast-conserving surgery; however, it remains challenging. Imaging-guided surgery provides precise detection of residual lesions and assists surgical resection. Herein, we described water-soluble melanin nanoparticles (MNPs) conjugated with cyclic Arg-Gly-Asp (cRGD) peptides for breast cancer photoacoustic imaging (PAI) and surgical navigation.

**Methods:**

The cRGD-MNPs were synthesised and characterized for morphology, photoacoustic characteristics and stability. Tumour targeting and toxicity of cRGD-MNPs were determined by using either breast cancer cells, MDA-MB-231 tumour-bearing mice or the FVB/N-Tg (MMTV-PyVT) 634Mul/J mice model. PAI was used to locate the tumour and guide surgical resection in MDA-MB-231 tumour-bearing mice.

**Results:**

The cRGD-MNPs exhibited excellent in vitro and in vivo tumour targeting with low toxicity. Intravenous administration of cRGD-MNPs to MDA-MB-231 tumour-bearing mice showed an approximately 2.1-fold enhancement in photoacoustic (PA) intensity at 2 h, and the ratio of the PA intensity at the tumour site to that in the surrounding normal tissue was 3.2 ± 0.1, which was higher than that using MNPs (1.7 ± 0.3). Similarly, the PA signal in the spontaneous breast cancer increased ~ 2.5-fold at 2 h post-injection of cRGD-MNPs in MMTV-PyVT transgenic mice. Preoperative PAI assessed tumour volume and offered three-dimensional (3D) reconstruction images for accurate surgical planning. Surgical resection following real-time PAI showed high consistency with histopathological analysis.

**Conclusion:**

These results highlight that cRGD-MNP-mediated PAI provide a powerful tool for breast cancer imaging and precise tumour resection. cRGD-MNPs with fine PA properties have great potential for clinical translation.

**Supplementary Information:**

The online version contains supplementary material available at 10.1007/s00259-021-05545-3.

## Introduction

Nearly half of patients with early breast cancer undergo breast-conserving surgery (BCS) with adjuvant radiation therapy [1]. BCS involves the removal of the primary tumour with a margin of surrounding normal breast tissue. Although BCS is the standard of care treatment for early breast cancer patients [2], a potential downside is the failure to achieve clean or negative margins with lumpectomy. In particular, 20–40% of patients require additional operative intervention by re-excision or even mastectomy [3]. Re-excision surgery increases the risk of complications (e.g. infection and unsatisfactory aesthetic results) and potentially postpones systemic treatment. It also increases the costs and burden of medical care and takes a toll on patients both physically and psychologically.

During BCS, surgeons typically use visual inspection and tactile feedback to determine the tumour location and set resection margins. However, it is difficult to discriminate tumour margins from surrounding normal tissue macroscopically. Frozen section and imprint cytology are applied clinically for intraoperative margin assessment. These methods have the potential to lower the rates of positive margins, but they are labour-intensive and time-consuming and have low sensitivity due to sampling rate limitations [4, 5]. Currently, several emerging imaging tools are being attempted for real-time margin assessment, including wire-guided localisation [6], intraoperative ultrasound (US) [7] and postoperative techniques (intraoperative specimen mammography/micro-computed tomography) [8]. Since the techniques mentioned above mainly focus on anatomical imaging and have limited tumour specificity, they have not been widely used for intraoperative margin assessment. Fluorescence-guided surgical navigation is a highly sensitive technique using the injection of a fluorescent contrast agent that is a promising intraoperative tool for precise tumour resection [9]. However, this technique is hampered by a limited penetration depth because of light scattering and signal attenuation [10].

Recently, photoacoustic imaging (PAI) has been developed as a novel imaging technology for biomedical applications. PAI detects optical absorption contrast acoustically via the photoacoustic (PA) effect, a physical phenomenon that converts absorbed optical energy into acoustic energy [11]. Based on endogenous contrast molecules (e.g. oxyhaemoglobin, deoxyhaemoglobin, lipid, or DNA-RNA), PAI has been used in the clinical trial to demonstrate its highly desirable capabilities for breast cancer imaging in vivo and ex vivo, particularly for assessing tumour margins macroscopically and microscopically [12–14]. However, intrinsic chromophores provide access to only a limited range of biological processes but low contrast. Hence, molecular PAI for breast cancer still requires a targeted contrast agent that can selectively bind to surface receptors on cancer cells or respond to the tumour microenvironment [11, 15].

Natural melanin is a group of biopigments with multifunctionality (i.e. ultraviolet protection, radical scavenging, and photothermal conversion) [16]. Due to its good intrinsic biocompatibility, natural melanin or synthetic melanin-like nanomaterials have been successfully developed as novel nano-bioplatforms in bioimaging, therapy, theranostics, and biosensing [17, 18]. As an endogenous PA contrasting agent, melanin was used to detect the metastatic status of ex vivo human melanoma sentinel lymph nodes by multispectral optoacoustic imaging [19]. The results showed an excellent correlation with the histological assessment of melanoma cell infiltration with 100% sensitivity and 62% specificity [19].

**Scheme 1:**
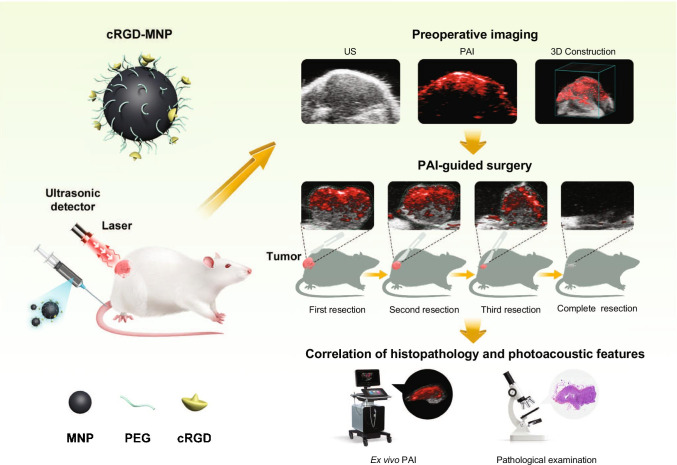
Workflow of cRGD-MNPs for intraoperative imaging-guided surgery by photoacoustic imaging

Integrin α_v_β_3_ is overexpressed on activated endothelial cells during angiogenesis as well as on many tumour cells within human breast carcinomas, participating in the tumorigenesis, invasiveness, and metastasis [20, 21]. Arg-Gly-Asp (RGD) tripeptide sequence for targeting integrin α_v_β_3_ has been well recognised and applied to develop the α_v_β_3_-specific tracer [22]. In this study, we conducted a comprehensive study of the feasibility of the PAI method for improving the detection and accurate removal of breast cancer using melanin nanoparticles (MNPs) conjugated with cyclic Arg-Gly-Asp (cRGD) (Scheme 1). Our data support the potential clinical application of cRGD-MNPs as a novel tumour-specific PA contrast agent for imaging-guided BCS.

## Materials and methods

### Reagents

Melanin was obtained from Sigma-Aldrich (Merck, Darmstadt, Germany). Sodium hydroxide, hydrochloric acid and NH_4_OH solution (28 wt%) were purchased from Sinopharm Chemical Reagent Co., Ltd (Shanghai, China). Amine-PEG_5000_-amine (NH_2_-PEG_5000_-NH_2_, 5 kDa) was obtained from Shanghai Aladdin Bio-Chem Technology Co., LTD (Shanghai, China). CellTiter 96 AQueous One Solution Cell Proliferation Assay (MTS) was purchased from Promega (Beijing, China).

### Cell culture

The human breast cancer cell line MDA-MB-231 and non-cancerous mammary epithelial cell line MCF-10A were purchased from Procell Life Science & Technology Co., Ltd. (Wuhan, China), and cells with passage number < 25 were used in the experiment. MDA-MB-231 cells and MCF-10A cells were cultured according to the vendors’ recommendations.

### Animals

All animal experiments were performed in compliance with the Guidelines for the Care and Use of Research Animals established by the Xiamen University Animal Studies Committee. Female BALB/c nude mice and BALB/c mice (4–6 weeks) were purchased from the Experimental Animal Centre of Xiamen University. FVB/N-Tg (MMTV-PyVT)634Mul/J transgenic mice were purchased from the Jackson Laboratory.

### Preparation of cRGD-MNPs

The cRGD-MNPs were obtained as previously described [23]. Briefly, melanin (2 mg/mL) was dissolved in NaOH aqueous solution (0.1 N) followed by the rapid addition of HCl aqueous solution (0.1 N) to a pH of 7.0 under sonication. The neutralised solution was centrifuged and washed with deionised water several times. The black solid of MNPs was obtained by freeze-drying. The MNP aqueous solution (1 mg/mL, pH = 9) was added dropwise into an NH_2_-PEG_5000_-NH_2_ aqueous solution. After vigorous stirring for 12 h, the PEG-modified MNPs were retrieved by centrifugation and washed several times to remove the unreacted NH_2_-PEG_5000_-NH_2_. The PEGylated MNPs (1 mg/mL, pH = 7.2) were incubated with 4-(N-maleimidomethyl)cyclohexane-1-carboxylic acid 3-sulfo-N-hydroxysuccinimide ester sodium salt (sulfo-SMCC) solution (1.2 mg in 36 μL of dimethyl sulfoxide) for 2 h at room temperature. The complexes were purified using a PD-10 column. The cyclic Arg-Gly-Asp-d-Phe-Cys peptide (cRGD) solution (120 μL of 5 mM in degassed water) (GL Biochem, Shanghai, China) was added to the purified MNPs with stirring for 24 h at 4 °C. The excess cRGD peptide was removed using a PD-10 column. The final cRGD-MNPs were filtered through a 0.22-μm filter.

### Characterisation of MNPs

Transmission electron microscopy images were recorded using a Talos F200s transmission electron microscope (Thermo Fisher Scientific, Waltham, USA) at an accelerating voltage of 100 kV. The MNP or cRGD-MNP aqueous solution was dropped onto a carbon-coated copper grid and air-dried. The ^1^H NMR spectra were recorded at 20 °C on a 400-MHz NMR spectrometer (Bruker) using deuterium oxide as the solvent. Zeta potentials were measured using a laser particle size analyser system (Zetasizer Nano ZS90, Malvern, UK). The absorption spectra were obtained using a Multiskan Spectrum Microplate Spectrophotometer (Thermo Fisher Scientific, Waltham, USA).

### PA signal of cRGD-MNPs

The solutions of MNPs and cRGD-MNPs (120 μM) were scanned for PAI using the Vevo LAZR-X photoacoustic imaging system (FujiFilm VisualSonics, Inc., Toronto, Canada) at different wavelengths ranging from 680 to 970 nm (interval = 5 nm) to detect the optimal excitation wavelength. Different concentrations (3.75–120 μM) of MNPs and cRGD-MNPs were dispersed in PBS and triggered by the optimal excitation wavelength to acquire the corresponding PA images. For photostability analysis, PA images of the MNP and cRGD-MNP solutions (120 μM) were obtained at different time points (0–7 days) using a 680-nm excitation.

### Cellular uptake of cRGD-MNPs

MDA-MB-231 cells (~ 3 × 10^5^ per well) were seeded in 6-well plates and cultured for 24 h. Then, the cells were incubated with 0.125 μM cRGD-MNPs for various times (1, 2, 4 or 8 h) and fixed with 4% paraformaldehyde (PFA) solution for 20 min. PAI of the MDA-MB-231 cells at different time points were performed at a wavelength of 680 nm using the Vevo LAZR-X system. For the concentration gradient experiment, MDA-MB-231 cells were incubated in medium containing the cRGD-MNPs (0.125–1 μM) for 4 h. To investigate the effect of cRGD on MNP uptake, MDA-MB-231 cells were treated for 4 h with medium containing 0.5 μM MNPs, PEG-MNPs or cRGD-MNPs, and then subjected to PAI analysis. To investigate the tumour targeting ability of cRGD-MNPs, MDA-MB-231 and MCF-10A cells (~ 3 × 10^5^ per well) were seeded in 6-well plates and incubated with 0.5 μM cRGD-MNPs alone or with excess cRGD peptide (100 μM) for 4 h.

### Preparation of Rho-MNPs and Rho-cRGD-MNPs

Rhodamine (30 μL, 5 mg/mL) was added to the MNPs or cRGD-MNPs solutions (1 mL, 2 mg/mL). After stirring at room temperature for 1 h, the solution was transferred to an ultrafiltration centrifuge tube (Millipore Amicon Ultra, 30 kDa) and centrifuged several times (4000 rpm, 15 min) to remove the free or unstable rhodamine, yielding the rhodamine-labelled MNPs (Rho-MNPs) and cRGD-MNPs (Rho-cRGD-MNPs).

### Location of cRGD-MNPs by confocal microscopy

MDA-MB-231 and MCF-10A cells (~ 1 × 10^5^ cells) were seeded in confocal dishes for 24 h. The Rho-cRGD-MNPs (0.5 μM) were added to the culture medium with or without free cRGD (100 μM) at 4 h. The cells were fixed with 4% PFA and counterstained with DAPI. The fluorescence images of the cells were collected using a confocal laser scanning microscope (FV1000, Olympus, Tokyo, Japan).

### Immunofluorescence staining

MDA-MB-231 cells (~ 1 × 10^5^/well) were incubated in cell slides for 24 h and treated with Rho-cRGD-MNPs or Rho-MNPs (0.5 μM) for 4 h. The cells were fixed with 4% PFA for 10 min, and blocked with PBST containing 1% BSA and 22.52 mg/mL glycine for 30 min. The cells were incubated with anti-integrin α_v_ antibody (1:500, Abcam, #ab179475) at 4 ℃ overnight, following by incubation with anti-rabbit antibody (1:1000, CST, #4412) for 1 h at room temperature. Finally, the cells were stained with DAPI and observed with a fluorescence microscope (Olympus, Tokyo, Japan).

### In vitro cytotoxicity assay

MDA-MB-231 cells (~ 5 × 10^3^/well) were incubated in 96-well plates for 24 h. Cells were then cultured in medium supplemented with the MNPs, PEG-MNPs or cRGD-MNPs (0.625, 1.25, 2.5, 5 and 10 μM) for 72 h. The MTS assay was performed following the vendors’ recommendations. Five replicates were conducted for each group.

### MDA-MB-231 breast cancer xenograft mouse model

MDA-MB-231 cells (~ 1 × 10^6^) were inoculated subcutaneously in the right flank of nude mice. When tumour volume reached 100 mm^3^, the tumour-bearing mice were subjected to in vivo PAI and biodistribution studies.

### In vivo tumour PAI in MDA-MB-231 tumour-bearing mice

To determine the optimal concentration of the MNPs for in vivo study, mice were randomly allocated into three groups and injected with different concentrations of cRGD-MNPs (50 μM, 100 μM or 200 μM, 200 μL). PA images were collected at different time points (pre, 1, 2, 4 or 12 h) using the Vevo LAZR-X at a wavelength of 680 nm, and the average PA intensities in the tumour regions were measured. To detect the tumour targeting capability of the cRGD-MNPs, MDA-MB-231 tumour-bearing mice were randomly divided into MNPs and cRGD-MNPs groups (*n* = 3 each group) and intravenously injected with the MNPs or cRGD-MNPs (100 μM, 200 μL), respectively. PA images were collected and analysed.

### In vivo blocking study of cRGD-MNPs

MDA-MB-231 tumour-bearing mice were randomly divided into two groups (each group *n* = 3) and injected with cRGD-MNPs or cRGD-MNPs (100 μM, 200 μL) with cRGD peptide (20 mg/kg). As for the blocking group, cRGD peptide was injected 15 min before cRGD-MNP injection. PA signals were collected at different time points (pre, 2 h).

### The biodistribution of MNPs and cRGD-MNPs in MDA-MB-231 tumour-bearing mice

To determine the biodistribution of the nanoprobes in vivo, MDA-MB-231 tumour-bearing mice (*n* = 3 each group) were intravenously injected with Rho-MNPs or Rho-cRGD-MNPs (100 μM, 200 μL). The major organs and tumours were harvested 2 h post-injection for ex vivo fluorescence imaging using the IVIS Lumina imaging system (PerkinElmer, Waltham, USA). The relative fluorescence intensities were measured.

### PAI and pathological analysis of spontaneous breast cancer in transgenic mice

The transgenic mouse model spontaneously develops invasive breast cancer in each mammary gland between 6 and 12 weeks of age. Mice with breast cancer (age 6–8 weeks, *n* = 6) were intravenously injected with the cRGD-MNPs (100 μM, 200 μL). US and PA images were obtained before injection and 2 h post-injection with excitation at 680 nm. In addition, the 4^th^ and 5^th^ mammary glands of the transgenic mice (age 8 weeks, *n* = 3) with breast cancer were divided into four sections (P1–4) for in vivo and ex vivo PAI at 2 h post-injection of cRGD-MNPs. After imaging, the mammary tissues were also analysed by histology.

### Melanin staining

Breast cancer tissues acquired from the transgenic mice after the injection of PBS or cRGD-MNPs were immersed in the Fontana–Masson solution (Servicebio, Wuhan, China), placed in the dark at 4℃ for 12–18 h. Next, the tissues were put into VG staining solution and stained for 1 min. Finally, the tissues were observed under a microscope (Olympus, Tokyo, Japan).

### PAI-guided breast cancer surgery on MDA-MB-231 xenograft mice

For surgical resection, cRGD-MNPs (100 μM, 200 μL) were intravenously injected into MDA-MB-231 tumour-bearing mice. Tumour profiles were detected using 3D PA/US imaging 2 h post-injection under 2% isoflurane anaesthesia. For simulated tumour surgical resection, the surgery procedure was performed in four steps: PAI-guided tumour detection, partial tumour resection (P1–3), PAI detection of the residual tumour, and re-resection of the tumour bed (P4). PA images were acquired both before and after each sequential removal of tumour. Each resection was about a 5–6 mm-long and 2–3-mm-wide piece of tissue. Excised tissue was stained with H&E staining. To detect the depth of PAI, PAI of tumours were performed under various thicknesses of chicken breast (1, 2, 3, 5, 7 mm).

### cRGD-MNP biosafety evaluation

BALB/c mice (4–6 weeks) were randomly divided into control group (saline, 200 μL) and cRGD-MNPs group (100 μM, 200 μL) (*n* = 15 each group). Body weight was monitored throughout the experiment. The mice were sacrificed on days 1, 7 and 30 post the injection. Blood samples and major organs (heart, liver, spleen, lung and kidney) were collected at above time points. The serum biochemical parameters were analysed, including aspartate aminotransferase (AST), alanine aminotransferase (ALT), blood urea nitrogen (BUN) and creatinine (CR). H&E staining of major organs was performed for histological analysis.

### Statistical analysis

Statistical analysis was performed using GraphPad Prism 7.0 (GraphPad Software Inc., San Diego, USA). The two-tailed paired *t*-test was used to compare the changes in photoacoustic signals. Data are presented the mean ± standard deviation. Significant differences between or among the groups are indicated as follows: ns for no significant difference, * for *p* < 0.05, ** for *p* < 0.01, and *** for *p* < 0.001.

## Results

### Synthesis and characterisation of cRGD-MNPs

**Fig. 1:**
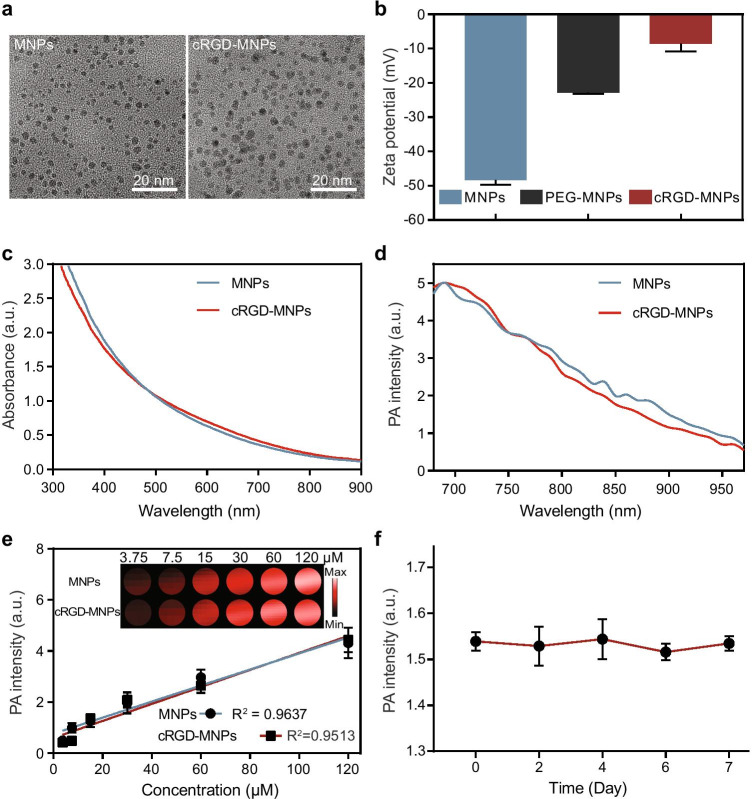
Characterisation of cRGD-MNPs. **a** Representative transmission electron microscopy images of MNPs (left) and cRGD-MNPs (right), scale bar: 20 nm. **b** Zeta potential of MNPs, PEG-MNPs and cRGD-MNPs in aqueous solution. **c** UV–vis-NIR spectra of MNPs and cRGD-MNPs. **d** PA spectra of MNPs and cRGD-MNPs. **e** The photoacoustic signal produced by MNPs and cRGD-MNPs was linear at concentrations from 3.75 to 120 μM. Upper panel: the original PA image. **f** PA signal intensities of cRGD-MNPs in PBS (pH = 7.4) stored for 1 week. Data are presented as the mean ± SD (*n* = 3)

The water-soluble MNPs were approximately 4 nm in size detected by transmission electron microscopy (Fig. 1a). Polyethylene glycol (PEG) chains were introduced into the MNPs for further biomodification, confirmed by ^1^H NMR spectra (Fig. S1a). Lastly, the PEG-MNPs were further modified with cRGD, which binds to tumour α_v_β_3_ integrin [24]. The diameter of the RGD-functionalised PEG-MNPs (cRGD-MNPs) increased to about 9 nm (Fig. 1a). With the modifications of PEG and cRGD, the surface potential of the cRGD-MNPs increased from − 48 to − 8.6 mV (Fig. 1b). MNPs have strong and broad optical absorbance and PA spectra in the wavelength range of visible light to the near-infrared region. The similar absorption and PA spectra between cRGD-MNPs and MNPs indicated that the biomodifications did not influence the absorption properties and intrinsic PA properties of melanin (Fig. 1c, d). Moreover, both the cRGD-MNPs and MNPs showed a linear relationship between concentration and absorbance intensity (*R*^2^ = 1 and 0.9999 for MNPs and cRGD-MNPs, respectively) (Fig. S1b, c, d) and PA intensity (*R*^2^ = 0.9637 and 0.9513 for MNPs and cRGD-MNPs, respectively) (Fig. S1e, f, Fig. 1e) at 680-nm excitation. In addition, the cRGD-MNPs exhibited excellent stability, with no attenuation of the PA signal intensity after storage for a week at 4 °C (Fig. 1f).

### Targeting ability and cytotoxicity of cRGD-MNPs in vitro

**Fig. 2:**
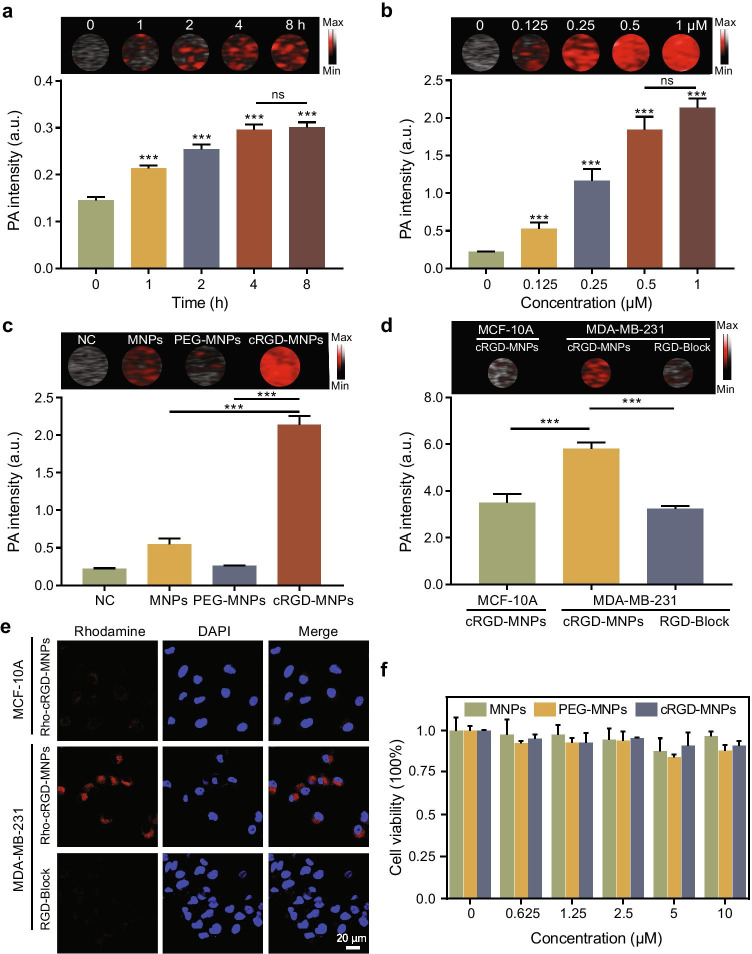
In vitro uptake and cytotoxicity of cRGD-MNPs. **a** PA images and signal intensities of MDA-MB-231 cells incubated with cRGD-MNPs for different times (0, 1, 2, 4 and 8 h). **b** PA images and signal intensities of MDA-MB-231 cells incubated with cRGD-MNPs at various concentrations (0, 0.125, 0.25, 0.5 and 1 μM). **c** PA images and signal intensities of MDA-MB-231 cells incubated with 0.5 μM MNPs, PEG-MNPs or cRGD-MNPs for 4 h. PBS was used as a negative control. **d** PA images and signal intensities of MCF-10A and MDA-MB-231 cells after incubation with cRGD-MNPs for 4 h with or without free cRGD blocking. **e** Confocal laser scanning micrographs of MCF-10A and MDA-MB-231 cells incubated with Rho-cRGD-MNPs for 4 h with or without free cRGD blocking. Scale bar: 20 µm. **f** MDA-MB-231 cell viability after incubation with MNPs, PEG-MNPs or cRGD-MNPs at gradient concentrations using standard MTS assay. Data are presented as the mean ± SD (*n* = 3), ***p < 0.001

As seen from the overlay images and quantitative results, the PA signal intensity of MDA-MB-231 cells increased when incubated with cRGD-MNPs from 1 to 4 h, and there was no statistical difference between the 4 h and 8 h time points (Fig. 2a). As the concentration of cRGD-MNPs increased, the uptake of the probe by the MDA-MB-231 cells also increased from 0.125 to 0.5 μM, and there was no statistical difference between 0.5 and 1 μM in PA signal intensity (Fig. 2b). The concentration of 0.5 μM cRGD-MNPs was selected for subsequent experiments. Moreover, MDA-MB-231 cells incubated with cRGD-MNPs showed stronger PA signal intensity than the cells incubated with MNPs or PEG-MNPs (Fig. 2c).

To confirm the ability of the cRGD-MNPs to target integrin α_v_β_3_, we determined their binding affinity using MDA-MB-231 and MCF-10A (a non-neoplastic breast cell line with low expression of α_v_β_3_) [25]. The PA intensity of MDA-MB-231 cells was stronger than that of MCF-10A cells after 4-h incubation with cRGD-MNPs (Fig. 2d). Additionally, when the cells were co-incubated with excess cRGD peptide, the PA signal intensity of the MDA-MB-231 cells became weaker (Fig. 2d). To further verify the targeting ability of cRGD-MNPs, rhodamine was conjugated with cRGD-MNPs or MNPs through intermolecular cross-linking and electron attraction. Obviously, red fluorescence signals were observed in MDA-MB-231 cells incubated with Rho-cRGD-MNPs, which was blocked by excess cRGD peptide (Fig. 2e). On the contrary, red fluorescence signals were hardly observed in MCF-10A cells under the same conditions (Fig. 2e). Moreover, immunofluorescence staining showed that Rho-cRGD-MNPs were co-localised with integrin α_v_ (Fig. S2). These data indicated that the tumour-specific targeting ability of cRGD-MNPs was mediated by integrin α_v_β_3_. Finally, data of cell viability showed low cytotoxicity for three nanoprobes (MNPs, PEG-MNPs or cRGD-MNPs), even at a high concentration up to 10 μM (Fig. 2f).

### In vivo cRGD-MNP tumour uptake and the biodistribution of fluorescence-labelled cRGD-MNPs

**Fig. 3:**
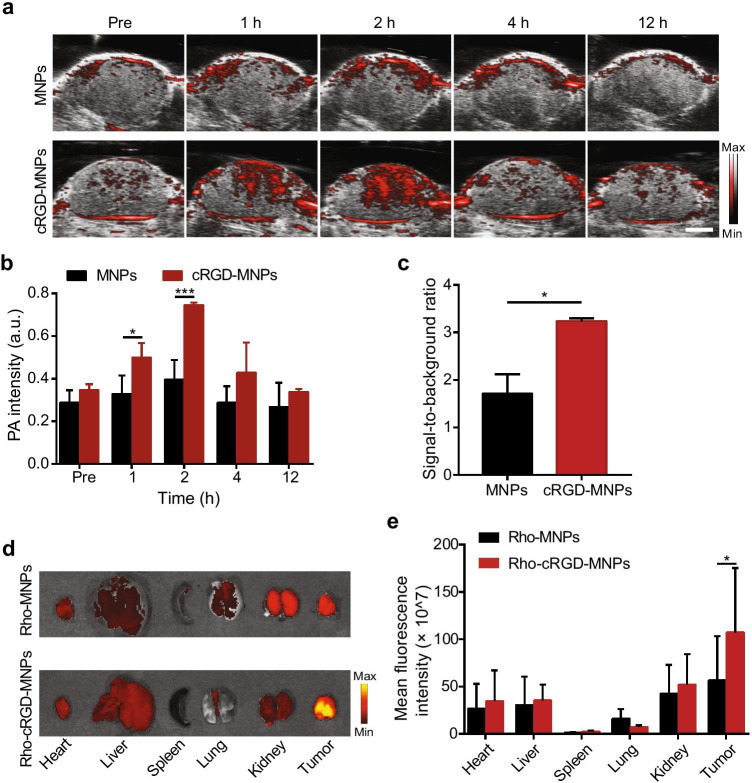
Tumour targeting and biodistribution of cRGD-MNPs using an MDA-MB-231 xenograft mouse model. **a** In vivo merged PA and US images of MDA-MB-231 tumour-bearing mice at different times (pre, 1, 2, 4 and 12 h) after intravenous injection of MNPs or cRGD-MNPs (100 μM, 200 μL). Scale bar: 2 mm. **b** Quantitative analysis of PA intensities of tumour sites at the different time points in **a**. **c** Tumour-to-background tissue ratios obtained at 2 h post-injection of MNPs or cRGD-MNPs. **d** The biodistribution of rhodamine-labelled MNPs or cRGD-MNPs in the heart, liver, spleen, lung, kidney and tumour from MDA-MB-231 tumour-bearing mice 2 h post-injection. **e** Quantitative analysis of the fluorescence intensities of the tissues in (**d**). Data are presented as the mean ± SD (*n* = 3); ***p < 0.001, *p < 0.05

Based on the PAI performance of the cRGD-MNPs in vitro, we performed tumour PAI in vivo. The in vivo PA signal intensity at the tumour site of MDA-MB-231 tumour-bearing mice at various concentrations of cRGD-MNPs (50, 100, and 200 μM) increased gradually and reached a peak 2 h post-injection (Fig. S3a). Moreover, the 100 μM and 200 μM groups exhibited similar PA intensities, which were higher than that observed for the 50 μM group (Fig. S3a). The tumour targeting capability of the cRGD-MNPs in living mice was further investigated by intravenous injection cRGD-MNPs into tumour-bearing mice using MNPs as a control. First, the tumour sites exhibited a higher PA signal 1 to 2 h after intravenous administration of cRGD-MNPs than MNPs in vivo (Fig. 3a, b). Next, the cRGD-MNP group had a significantly higher signal-to-background ratio (tumour vs surrounding normal tissue) than the MNP group 2 h post-injection (3.2 ± 0.1 vs 1.7 ± 0.3, *p* < 0.05) (Fig. 3c). In addition, the PA signal increased approximately 2.1-fold 2 h post-injection compared to pre-injection (Fig. S3b). To confirm that the tumour accumulation in vivo is driven by a specific ligand/receptor interaction, the blocking study was performed. As shown in Fig. S4, the PA signal of cRGD blocking group was significantly reduced at 2 h post-injection of cRGD-MNPs. These results indicated that cRGD-MNPs accumulated more in tumours than in normal tissue due to the targeting ability of cRGD peptide and presented clearer tumour contrast.

We also used fluorescence imaging to track the biodistribution of Rho-cRGD-MNPs and Rho-MNPs at 2 h post-injection (Fig. 3d). The ex vivo fluorescence images and fluorescence intensities of the major organs and tumours suggested more effective accumulation of Rho-cRGD-MNPs into tumour tissue than of Rho-MNPs (Fig. 3d, e).

### In vivo cRGD-MNP PAI in MMTV-PyVT transgenic mice

**Fig. 4:**
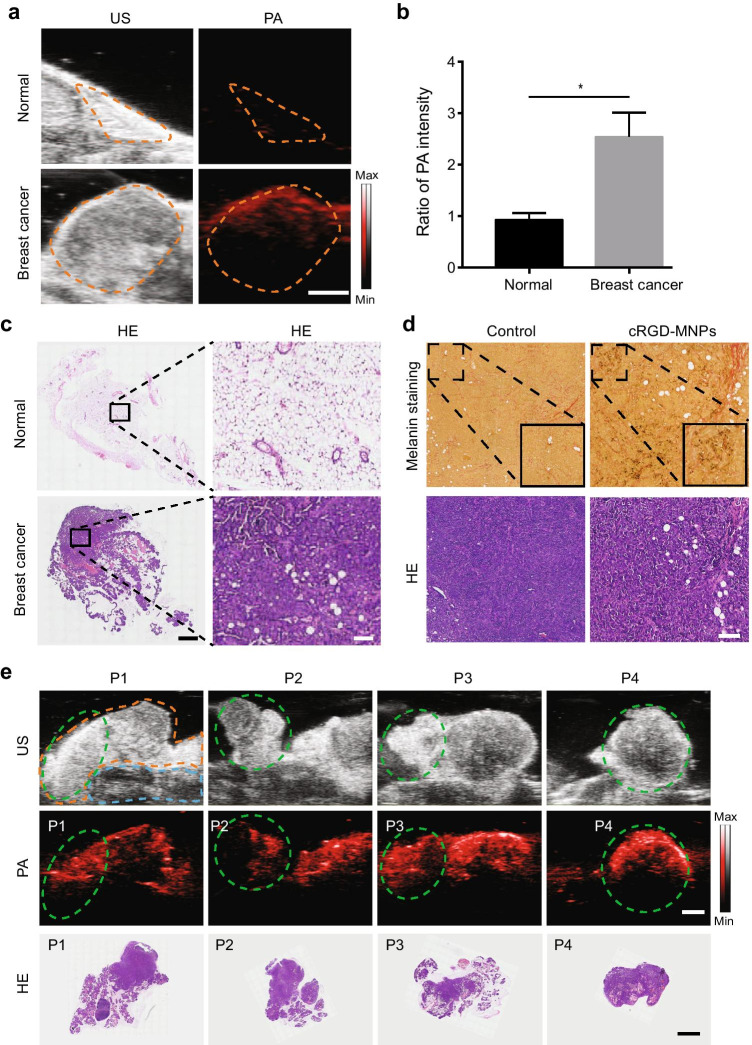
PAI for mammary glands containing spontaneous breast cancer in MMTV-PyVT transgenic mice. **a** Representative US and PA images of cRGD-MNP accumulation in mice with normal mammary glands or breast cancer. Scale bar: 2 mm. **b** The ratio of PA intensity of breast tumours or normal mammary glands in transgenic mice 2 h post-injection of cRGD-MNPs to 0 h. **c** Histopathological examination of the tissues from **a**. Scale bar: 2 mm (left) and 200 µm (right). **d** Fontana–Masson staining of ex vivo tumour tissues at 2 h post-injection of cRGD-MNPs or not. Black particles representing the cRGD-MNPs. The image in the lower right corner (black square) is an enlarged image of the upper left corner area. Scale bar: 200 µm. **e** US (top), PA (middle) and histological (bottom) images of the 4^th^ and 5^th^ mammary glands in an 8-week-old MMTV-PyVT mouse. The enlarged mammary glands and different regional tissues (P1–4) are outlined with dotted orange and green lines, respectively. The dotted blue line outlines the tissues inferior to the mammary glands. Scale bar: 2 mm. Data are presented as the mean ± SD (*n* = 3); *p < 0.05

To fully evaluate the PAI performance of cRGD-MNPs, we used the FVB/N-Tg(MMTV-PyVT)634Mul/J spontaneous breast cancer model that closely recapitulates human disease [26]. The transgenic mice underwent PAI before or 2 h post-injection of cRGD-MNPs (Fig. 4a). Compared to the imaging signal before administration, the PA signal intensities of the mammary glands containing tumour increased 2.5 ± 0.3-fold (Fig. 4b) at 2 h post-injection. In contrast, the PA signal intensity of the normal mammary glands showed no increase (0.9 ± 0.1-fold, Fig. 4b). The pathological status of the mammary glands was determined by H&E staining (Fig. 4c). Furthermore, melanin staining confirmed the presence of the MNPs in the tumour tissue after the injection of the cRGD-MNPs (Fig. 4d). Altogether, these results indicated that cRGD-MNPs could provide a high signal intensity at the tumour site and distinguish between normal mammary glands and breast tumours.

In order to further assess the feasibility of using the cRGD-MNPs to detect a tumour, segmented tumour PAI was performed in MMTV-PyVT transgenic mice with breast cancer (Fig. 4e). The 4^th^ and 5^th^ mammary glands of the transgenic mice were surgically divided into four segments (P1–4), and PA imaging showed complete and intense enhancement of each part of the tumour. Ex vivo tissues were imaged using the PAI system, and the tissue signal distribution (Fig. S5a) was consistent with that observed in vivo and also correlated with the pathological examination. The PA images demonstrated an improved contrast profile for the detection of breast cancer with the cRGD-MNPs.

### PAI-guided resection of breast cancer using cRGD-MNPs

**Fig. 5:**
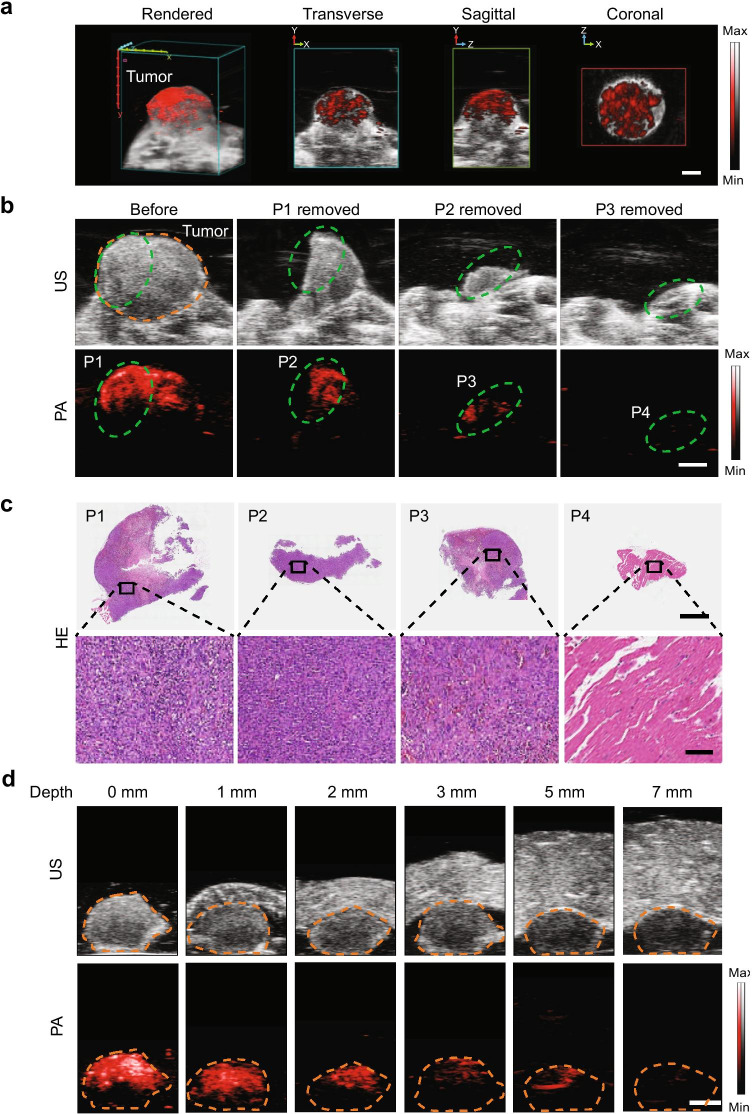
PAI-guided tumour resection in MDA-MB-231 tumour-bearing mice. **a** Preoperative 3D reconstruction (including axial, sagittal and coronal images) at 2 h post-injection of cRGD-MNPs. Scale bar: 2 mm. **b** Anatomical US (top) and PA (bottom) images showing the tumour region (dotted orange line) in MDA-MB-231 tumour-bearing mice in vivo. The tissues to be resected (P1–4) is highlighted with a green dashed circle. Scale bar: 2 mm. **c** Histological images (top) of resected tissues. Scale bar: 2 mm. Enlarged images of regions marked with black boxes are also shown (bottom). Scale bar: 100 μm. **d** PA and US images of tumour tissues covered by chicken breasts of different thicknesses under 680-nm laser excitation. Scale bar: 2 mm

cRGD-MNP-based PAI was evaluated for tumour detection, delineation, and PAI-guided resection. First, the tumours of MDA-MB-231 tumour-bearing mice were examined by PAI preoperatively. The reconstructed three-dimensional (3D) images showed the spatial distribution of the cRGD-MNPs at the tumour site, which provided the general profile of a tumour for the development of a surgical plan (Fig. 5a). From the PA signals of the cRGD-MNPs, the size and position of the MDA-MB-231-tumour could be defined. Next, we investigated whether the nanoprobes could guide intraoperative tumour resection. In this experiment, MDA-MB-231 tumour-bearing mice underwent consecutive tumour resections 2 h post-injection of cRGD-MNPs. US images depicted representative tumours xenograft (Fig. 5b). The tumour showed strong PA signal before the resection. Then, partial resection was performed under PAI guidance. The resected tissues (P1–4) for each step are shown in Fig. 5b. After removal of most of the tumour, the remaining tumour (about 2 mm wide) was still visible by PAI. Finally, the remaining tumour was resected until no obvious PA signal was detectable. For comparison, tissues with negative PA signals on the tumour bed (P4) were also excised for subsequent pathology examination. The ex vivo tissue PA signal distribution was consistent with that obtained in vivo (Fig. S5b). Pathological examination showed that the tumour had been completely removed, and the tissues with a negative PA signal on the tumour bed were free of residual tumour (Fig. 5c). To detect the depth of PAI, we covered the tumours with chicken breasts of various thicknesses and found that the maximum imaging depth was up to 5 mm (Fig. 5d). Taken together, cRGD-MNP-based PAI could effectively detect tumours, identify residual masses, and guide surgical resection.

### In vivo cRGD-MNP biosafety

The in vivo biocompatibility was further evaluated in mice for prolonged durations (1, 7 or 30 days) after intravenous administration of cRGD-MNPs. Saline served as a negative control. No considerable body weight loss was observed for the cRGD-MNPs and control groups over 30 days, indicating that cRGD-MNPs had no significant side effects in mice (Fig. S6a). The serum biochemical analysis, which included liver function (ALT, AST) and kidney function (BUN, CR) markers, showed negligible variations between the cRGD-MNPs and control groups, indicating no detectable toxicity shortly after or a relatively long time after exposure (Fig. S6b). H&E staining of the major organs (heart, liver, spleen, lung and kidney) at different time points showed no significant acute or chronic physiological toxicity compared to the control group (Fig. S6c). These results indicated that the cRGD-MNPs had high in vivo biocompatibility.

## Discussion

It remains a significant challenge in BCS to acquire clean margins during primary surgery due to the lack of precision in localising tumours and the inaccuracy of tumour excision by visual inspection and tactile feedback. Imaging-guided surgery is gaining increasing importance in the operating room. It can help detect microscopic tumours or residual lesions that are readily missed during surgery and guide intraoperative surgical margin assessment [27]. Thus, this approach could potentially improve patient outcome following oncologic surgery. In the present study, we demonstrated, for the first time, that the use of cRGD-MNPs as a targeted PA contrast has the potential to locate tumours and offer 3D reconstruction of the breast cancer for surgical planning. Although the utility of cRGD-MNP-mediated PAI or photothermal therapy has been previously demonstrated in animals [17], our study further translates these findings to imaging-guided oncologic surgery, showing the feasibility of PAI using cRGD-MNPs for imaging-guided resections of breast cancer.

Melanin is a naturally occurring biopolymer present in many organisms, particularly in the skin and hair. It has a good intrinsic photoacoustic property. By mimicking natural melanin, recent studies have demonstrated that melanin-based nanoparticles could serve as a multimodality nanoplatform for molecular imaging [16, 28, 29]. As in previous study [23], we prepared artificial ultrasmall MNPs and modified them by conjugating them with cRGD peptides. cRGD have a high affinity for integrin α_v_β_3_. Consistent with previous findings [30, 31], our results demonstrated a higher tumour targeting capability of cRGD-MNPs compared to MNPs using MDA-MB-231 tumour-bearing mice, indicating that cRGD-MNPs could specifically target breast cancer xenografts, possibly via the binding to integrin α_v_β_3_. Moreover, mammary glands of FVB/N-Tg (MMTV-PyVT)634Mul/J transgenic mice containing carcinoma showed excellent PA intensity enhancement after the administration of cRGD-MNPs compared to the normal mammary gland tissue. These data further indicated that cRGD-MNPs could serve as a good contrast for breast cancer-specific PAI.

MNPs have been used in diverse biomedical applications, such as imaging, controlled drug release, bioengineering and bioelectronics, antioxidant applications and theranostics [32]. Based on the dramatic in vivo PAI properties of the cRGD-MNPs in this study, we used the nanoprobes for PAI-guided surgical navigation. To simulate PAI-guided consecutive resections in a small animal model, we sequentially removed breast tumour tissue using the real-time guidance of PAI, and observed a good correlation between PAI of the resected tissues and histology results. In addition, we found that the tumour, when artificially placed in deep tissue, could be clearly imaged and discriminated with a depth of up to 5 mm. A previous study reported the application of PAI with endogenous melanin as contrast for the resection of B16 melanoma liver metastasis [33]. Hepatic melanoma in vivo as small as 400 µm could be detected at a depth of up to 7 mm and precisely resected using PAI guidance, demonstrating the advantages of PA (i.e. high resolution, high sensitivity, deep penetration and early detection of hepatic micrometastasis). In a clinical study, PAI-guided pathological evaluation improved the detection rate of metastases compared to the standard protocol in excising sentinel lymph nodes in patients with melanoma (22.9% vs 14.2%) [19]. Taken together with the results of the present study, PAI using melanin or melanin-based nanoparticles could offer a rapid and effective tool for non-invasive detection of small tumour with a certain depth.

In addition to melanin nanoparticles, several exogenous contrast agents have been used for photoacoustic surgical navigation in animals, including gold nanoparticles [34, 35], superparamagnetic iron oxide [36], dye-based agents [37, 38], and carbon-based nanomaterials [39]. All these agents could generate non-invasive PA contrast enhancement when stimulated by laser irradiation at specific wavelengths. However, clinical translation of these agents is prohibitive because of biosafety issues, poor biodegradability, low photostability and unclear biocompatibility [40, 41]. In the present study, the biocompatibility and biosafety of cRGD-MNPs were systematically evaluated both in vitro and in vivo. As reported previously, the strengths of melanin-like nanomaterials include good biocompatibility and long-term photostability [42, 43], prompting us to explore their biomedical applications, particularly for in vivo imaging. Our findings demonstrated that cRGD-MNPs represent a promising contrast agent for further clinical translation.

However, this study has a few limitations. First, only PAI alone was applied to surgical navigation. As previous study reported [23], MNPs are an active platform to simplify the assembling of different imaging moieties, such as positron emission tomography and magnetic resonance imaging. Thus, complementary use of multimodality imaging is promising not only for accurate tumour imaging but also for guiding tumour resection. Further research efforts should be devoted to precise, targeted tumour multimodality imaging. Second, the current methodology using MNPs as a contrast agent is unsuitable for deep tissue imaging in the human body. Therefore, further improvements of the imaging agents are needed to increase the imaging depth.

In this report, we demonstrated the feasibility of PA augmented by the systemic delivery of cRGD-MNPs to provide preoperative 2D or 3D PA images of tumours for precise surgical planning, and guide initial and subsequent resections of breast cancer tumour mass. Prospectively, PAI using cRGD-MNPs is expected to be evaluated in breast cancer patients for surgical navigation in the near future.

## Supplementary Information

Below is the link to the electronic supplementary material.

Supplementary file1 (DOCX 3845 KB)

## Data Availability

The datasets generated during and/or analysed during the current study are available from the corresponding authors on reasonable request.

## References

[1] DeSantis CE, Ma J, Gaudet MM, Newman LA, Miller KD, Goding Sauer A (2019). Breast cancer statistics, 2019. CA Cancer J Clin.

[2] Darby S, McGale P, Correa C, Taylor C, Arriagada R, Clarke M (2011). Effect of radiotherapy after breast-conserving surgery on 10-year recurrence and 15-year breast cancer death: meta-analysis of individual patient data for 10,801 women in 17 randomised trials. Lancet.

[3] Pleijhuis RG, Graafland M, de Vries J, Bart J, de Jong JS, van Dam GM (2009). Obtaining adequate surgical margins in breast-conserving therapy for patients with early-stage breast cancer: current modalities and future directions. Ann Surg Oncol.

[4] Esbona K, Li Z, Wilke LG (2012). Intraoperative imprint cytology and frozen section pathology for margin assessment in breast conservation surgery: a systematic review. Ann Surg Oncol.

[5] Pradipta AR, Tanei T, Morimoto K, Shimazu K, Noguchi S, Tanaka K (2020). Emerging technologies for real-time intraoperative margin assessment in future breast-conserving surgery. Adv Sci (Weinh).

[6] Langhans L, Tvedskov TF, Klausen TL, Jensen M-B, Talman M-L, Vejborg I (2017). Radioactive seed localization or wire-guided localization of nonpalpable invasive and in situ breast cancer: a randomized, multicenter, open-label trial. Ann Surg.

[7] Ahmed M, Douek M (2013). Intra-operative ultrasound versus wire-guided localization in the surgical management of non-palpable breast cancers: systematic review and meta-analysis. Breast Cancer Res Treat.

[8] Qiu S-Q, Dorrius MD, de Jongh SJ, Jansen L, de Vries J, Schröder CP (2018). Micro-computed tomography (micro-CT) for intraoperative surgical margin assessment of breast cancer: a feasibility study in breast conserving surgery. Eur J Surg Oncol.

[9] Hernot S, van Manen L, Debie P, Mieog JSD, Vahrmeijer AL (2019). Latest developments in molecular tracers for fluorescence image-guided cancer surgery. Lancet Oncol.

[10] Nguyen QT, Tsien RY (2013). Fluorescence-guided surgery with live molecular navigation–a new cutting edge. Nat Rev Cancer.

[11] Wang LV, Yao J (2016). A practical guide to photoacoustic tomography in the life sciences. Nat Methods.

[12] Li R, Lan L, Xia Y, Wang P, Han LK, Dunnington GL, et al. High-speed intraoperative assessment of breast tumor margins by multimodal ultrasound and photoacoustic tomography. Med Devices Sens. 2018;1. 10.1002/mds3.10018. 10.1002/mds3.10018 PMC6703831 31435620

[13] Wong TTW, Zhang R, Hai P, Zhang C, Pleitez MA, Aft RL (2017). Fast label-free multilayered histology-like imaging of human breast cancer by photoacoustic microscopy. Sci Adv.

[14] Li R, Wang P, Lan L, Lloyd FP, Goergen CJ, Chen S (2015). Assessing breast tumor margin by multispectral photoacoustic tomography. Biomed Opt Express.

[15] Weber J, Beard PC, Bohndiek SE (2016). Contrast agents for molecular photoacoustic imaging. Nat Methods.

[16] Park J, Moon H, Hong S (2019). Recent advances in melanin-like nanomaterials in biomedical applications: a mini review. Biomater Res.

[17] Caldas M, Santos AC, Veiga F, Rebelo R, Reis RL, Correlo VM (2020). Melanin nanoparticles as a promising tool for biomedical applications - a review. Acta Biomater.

[18] Fu Q, Zhu R, Song J, Yang H, Chen X (2019). Photoacoustic imaging: contrast agents and their biomedical applications. Adv Mater (Deerfield Beach, Fla).

[19] Stoffels I, Morscher S, Helfrich I, Hillen U, Leyh J, Lehy J (2015). Metastatic status of sentinel lymph nodes in melanoma determined noninvasively with multispectral optoacoustic imaging. Sci Transl Med.

[20] Pignatelli M, Cardillo MR, Hanby A, Stamp GW (1992). Integrins and their accessory adhesion molecules in mammary carcinomas: loss of polarization in poorly differentiated tumors. Hum Pathol.

[21] Gasparini G, Brooks PC, Biganzoli E, Vermeulen PB, Bonoldi E, Dirix LY (1998). Vascular integrin alpha(v)beta3: a new prognostic indicator in breast cancer. Clin Cancer Res.

[22] Schottelius M, Laufer B, Kessler H, Wester HJ (2009). Ligands for mapping alphavbeta3-integrin expression in vivo. Acc Chem Res.

[23] Fan Q, Cheng K, Hu X, Ma X, Zhang R, Yang M (2014). Transferring biomarker into molecular probe: melanin nanoparticle as a naturally active platform for multimodality imaging. J Am Chem Soc.

[24] Nieberler M, Reuning U, Reichart F, Notni J, Wester HJ, Schwaiger M, et al. Exploring the role of RGD-recognizing integrins in cancer. Cancers. 2017;9. 10.3390/cancers9090116. 10.3390/cancers9090116 PMC5615331 28869579

[25] Veneti E, Tu RS, Auguste DT (2016). RGD-targeted liposome binding and uptake on breast cancer cells is dependent on elastin linker secondary structure. Bioconj Chem.

[26] Shishido SN, Delahaye A, Beck A, Nguyen TA (2014). The anticancer effect of PQ1 in the MMTV-PyVT mouse model. Int J Cancer.

[27] Mondal SB, O'Brien CM, Bishop K, Fields RC, Margenthaler JA, Achilefu S (2020). Repurposing molecular imaging and sensing for cancer image-guided surgery. J Nucl Med.

[28] Liu H, Yang Y, Liu Y, Pan J, Wang J, Man F (2020). Melanin-like nanomaterials for advanced biomedical applications: a versatile platform with extraordinary promise. Adv Sci (Weinh).

[29] Zhang L, Sheng D, Wang D, Yao Y, Yang K, Wang Z (2018). Bioinspired multifunctional melanin-based nanoliposome for photoacoustic/magnetic resonance imaging-guided efficient photothermal ablation of cancer. Theranostics.

[30] Zhang J, Mao F, Niu G, Peng L, Lang L, Li F (2018). Ga-BBN-RGD PET/CT for GRPR and integrin αβ imaging in patients with breast cancer. Theranostics.

[31] Beer AJ, Niemeyer M, Carlsen J, Sarbia M, Nährig J, Watzlowik P (2008). Patterns of alphavbeta3 expression in primary and metastatic human breast cancer as shown by 18F-Galacto-RGD PET. J Nucl Med.

[32] Hong Z-Y, Feng H-Y, Bu L-H. Melanin-based nanomaterials: the promising nanoplatforms for cancer diagnosis and therapy. Nanomedicine. 2020:102211. 10.1016/j.nano.2020.102211. 10.1016/j.nano.2020.102211 32320736

[33] Yu Q, Huang S, Wu Z, Zheng J, Chen X, Nie L (2019). Label-free visualization of early cancer hepatic micrometastasis and intraoperative image-guided surgery by photoacoustic imaging. J Nucl Med.

[34] Kircher MF, de la Zerda A, Jokerst JV, Zavaleta CL, Kempen PJ, Mittra E (2012). A brain tumor molecular imaging strategy using a new triple-modality MRI-photoacoustic-Raman nanoparticle. Nat Med.

[35] Guan T, Shang W, Li H, Yang X, Fang C, Tian J (2017). From detection to resection: photoacoustic tomography and surgery guidance with indocyanine green loaded gold nanorod@liposome core-shell nanoparticles in liver cancer. Bioconjug Chem.

[36] Thawani JP, Amirshaghaghi A, Yan L, Stein JM, Liu J, Tsourkas A. Photoacoustic-guided surgery with indocyanine green-coated superparamagnetic iron oxide nanoparticle clusters. Small (Weinheim an der Bergstrasse, Germany). 2017;13. 10.1002/smll.201701300. 10.1002/smll.201701300 PMC5884067 28748623

[37] Wilson KE, Bachawal SV, Willmann JK (2018). Intraoperative resection guidance with photoacoustic and fluorescence molecular imaging using an anti-B7-H3 antibody-indocyanine green dual contrast agent. Clin Cancer Res.

[38] Han Z, Shang W, Liang X, Yan H, Hu M, Peng L (2019). An innovation for treating orthotopic pancreatic cancer by preoperative screening and imaging-guided surgery. Mol Imag Biol.

[39] De la Zerda A, Zavaleta C, Keren S, Vaithilingam S, Bodapati S, Liu Z (2008). Carbon nanotubes as photoacoustic molecular imaging agents in living mice. Nat Nanotechnol.

[40] Zhang X-D, Wu D, Shen X, Liu P-X, Yang N, Zhao B (2011). Size-dependent in vivo toxicity of PEG-coated gold nanoparticles. Int J Nanomed.

[41] Bussy C, Methven L, Kostarelos K (2013). Hemotoxicity of carbon nanotubes. Adv Drug Del Rev.

[42] Liu Y, Ai K, Liu J, Deng M, He Y, Lu L (2013). Dopamine-melanin colloidal nanospheres: an efficient near-infrared photothermal therapeutic agent for in vivo cancer therapy. Adv Mater.

[43] Liu X, Cao J, Li H, Li J, Jin Q, Ren K (2013). Mussel-inspired polydopamine: a biocompatible and ultrastable coating for nanoparticles in vivo. ACS Nano.

