# Supplementary material for: RGD-functionalised melanin nanoparticles for intraoperative photoacoustic imaging-guided breast cancer surgery
Source: Eur J Nucl Med Mol Imaging. 2021 Sep 10;49(3):847–60. doi: 10.1007/s00259-021-05545-3 (PMC8803813; doi:10.1007/s00259-021-05545-3)
Supplement: Supplementary file 1 — Supplementary file1 (DOCX 3845 KB) [file 259_2021_5545_MOESM1_ESM.docx]

Supplementary Information for

**RGD-functionalized melanin nanoparticles for intraoperative photoacoustic imaging-guided breast cancer surgery**


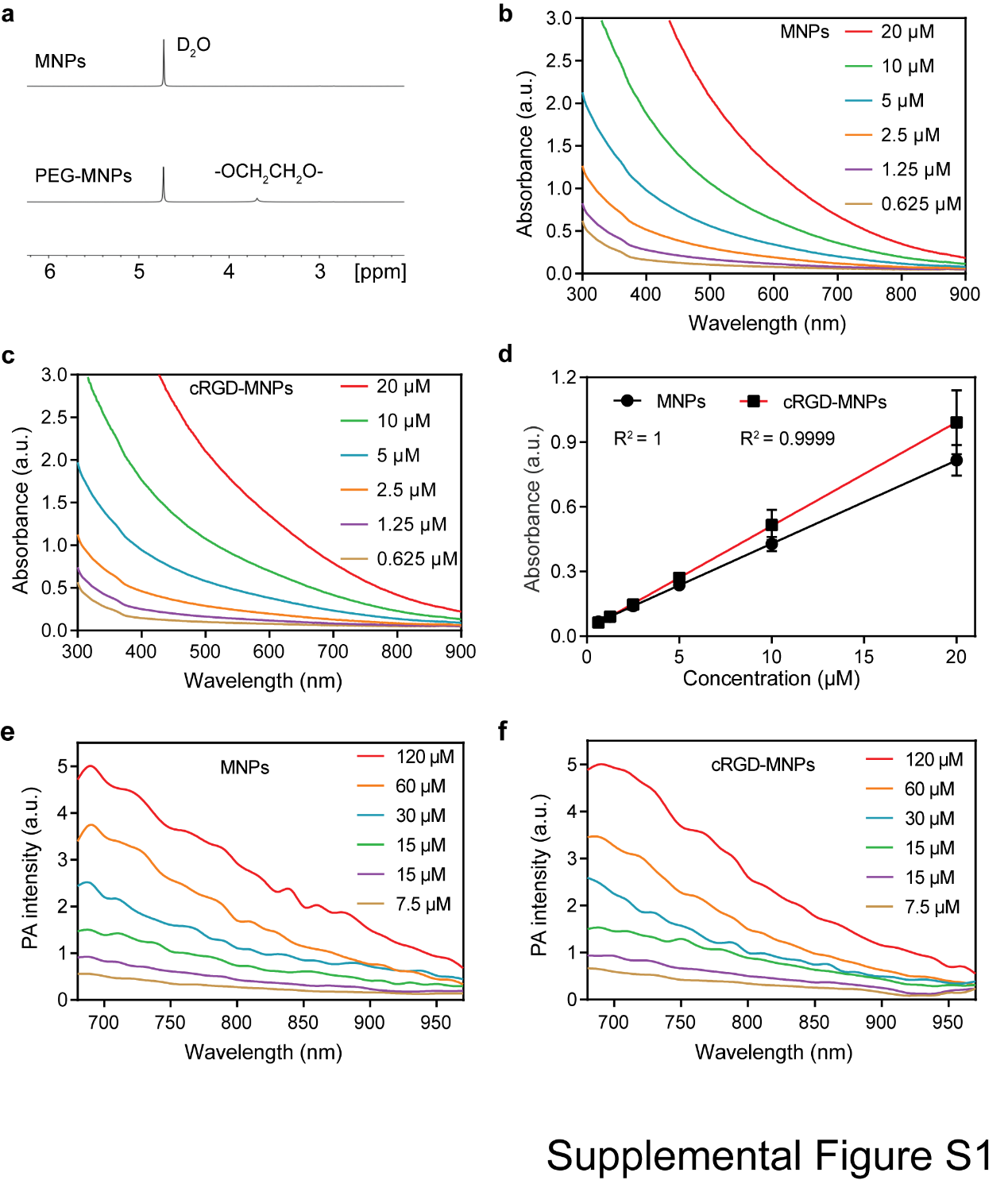


**Fig. S1 Characterization of MNPs, PEG-MNPs and cRGD-MNPs. a** ^1^H NMR spectra of MNPs and PEG-MNPs in D_2_O. **b** Absorption spectra of MNPs at different concentrations. **c** Absorption spectra of cRGD-MNPs at different concentrations. **d** Absorption intensities as a function of MNP or cRGD-MNP concentrations in PBS. **e** PA spectra of MNPs at various concentrations. **f** PA spectra of cRGD-MNPs at various concentrations.


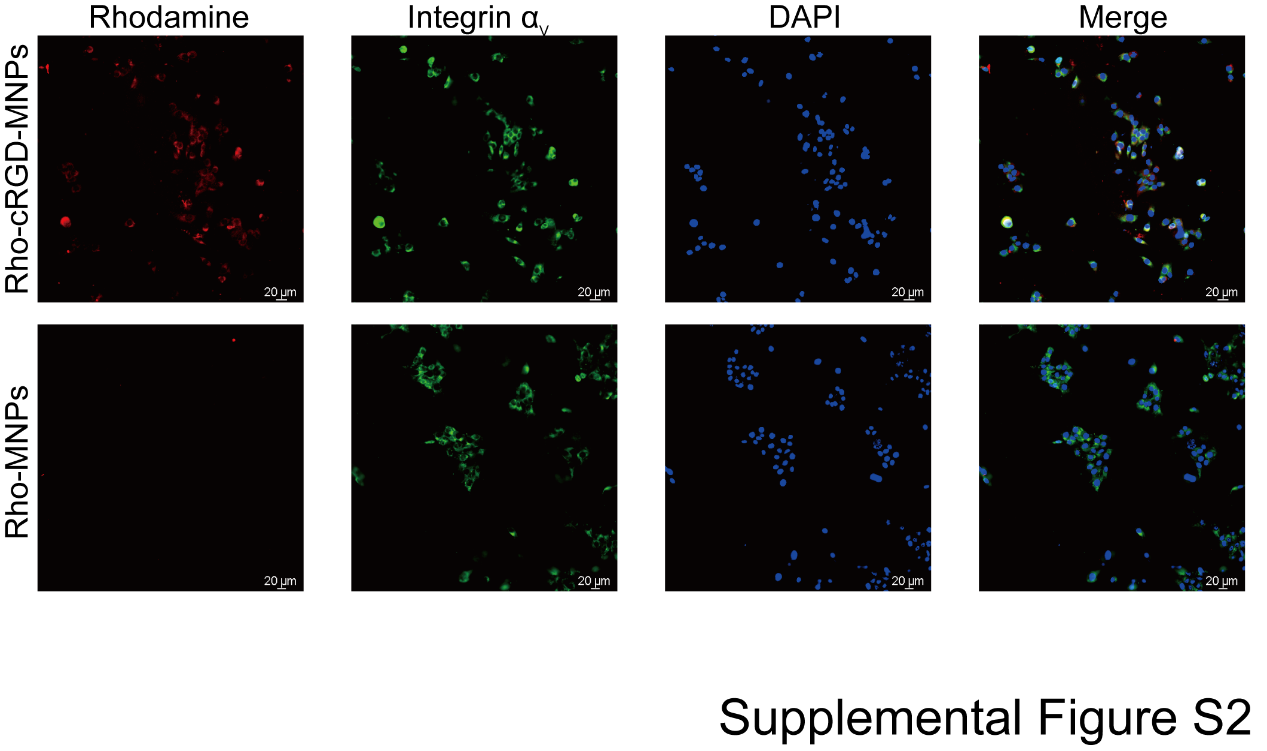


**Fig. S2 Co-localization of the cRGD-MNPs with integrin α_v_ in MDA-MB-231 cells.** MDA-MB-231 cells were incubated with Rho-cRGD-MNPs or Rho-MNPs for 4 h, followed by Immunofluorescence assay to verify the co-localization of probe and integrin α_v_ (red: Rhodamine, green: integrin α_v_, blue: DAPI). scale bar: 20 μm.


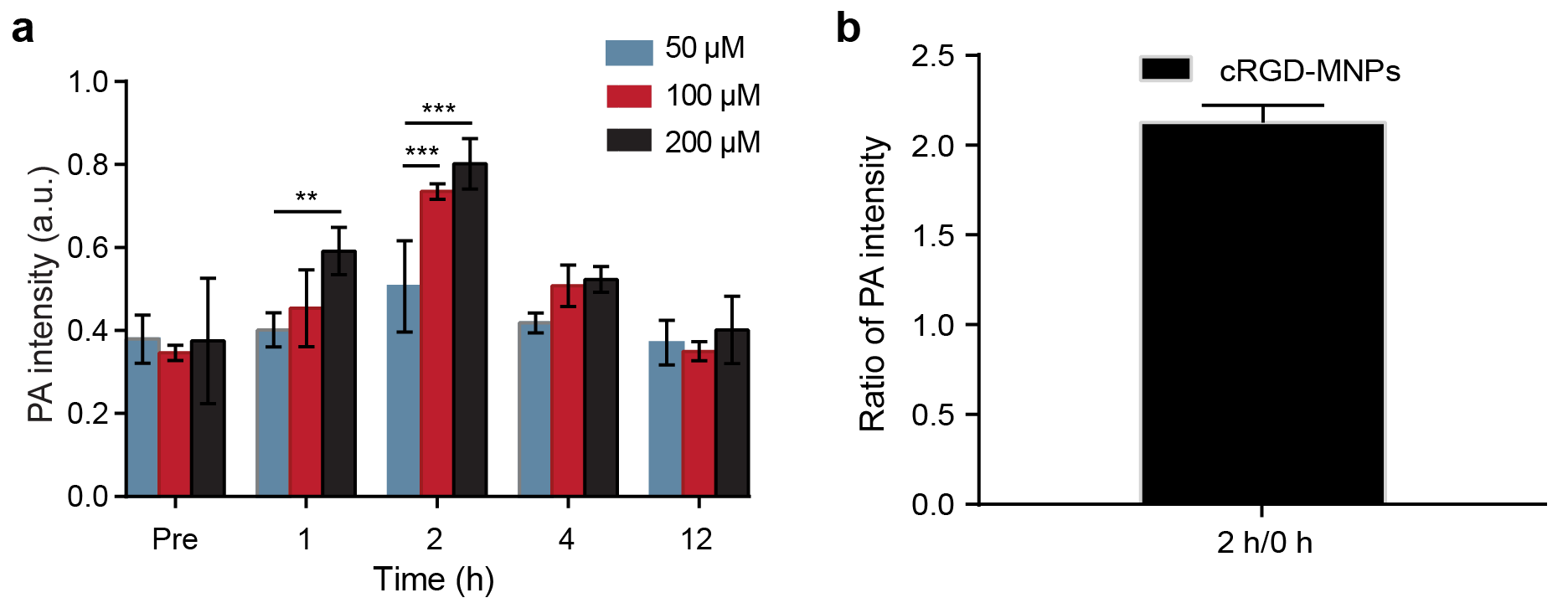


**Fig. S3 PAI of MDA-MB-231 tumours with different concentration of cRGD-MNPs. a** PA signal intensity of the tumour region in MDA-MB-231 tumour-bearing mice at various time points (0, 1, 2, 4 and 12 h) after intravenous injection of various concentration of cRGD-MNPs (50, 100, and 200 μM). **b** Quantitative analysis of enhanced PA signal of MDA-MB-231 tumour 2 h post-injection of cRGD-MNPs (100 μM, 200 μL) compared to 0 h. Data are presented as the mean ± SD (n = 3), *** *p* < 0.001, ** *p* < 0.01.


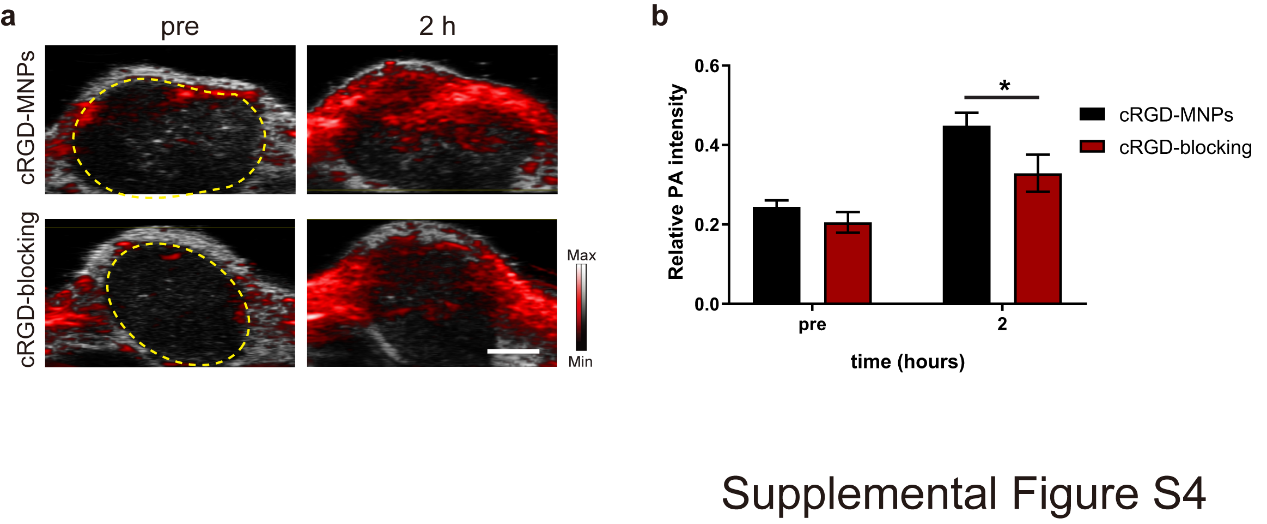


**Fig. S4 *In vivo* blocking study of cRGD-MNPs. a** *In vivo* merged PA and US images of MDA-MB-231 tumour before and after intravenous injection of cRGD-MNPs or cRGD-MNPs with cRGD blocking. **b** Quantitative analysis of PA intensities of tumour sites at the different time points of (**a**).

* *p* < 0.05.


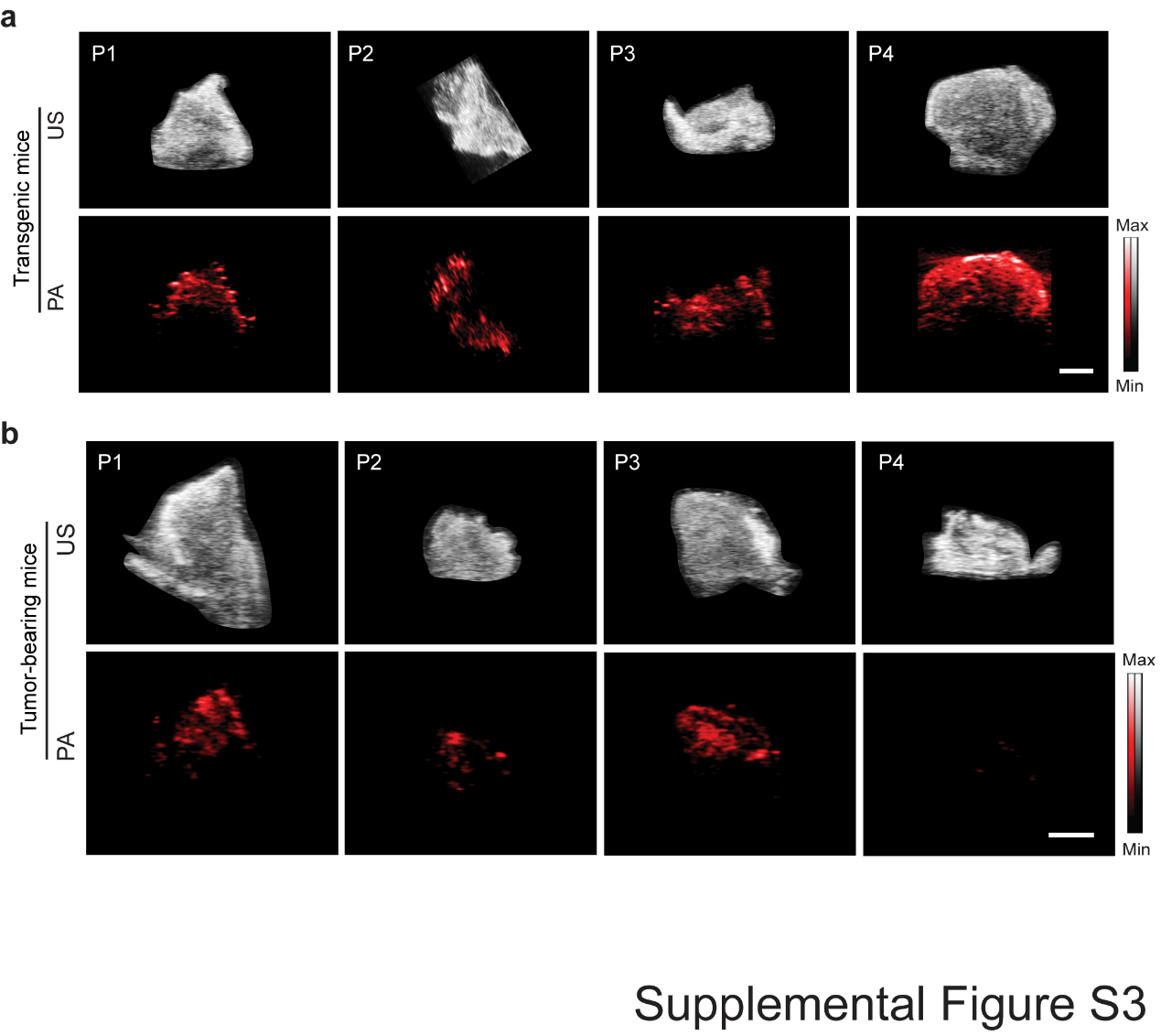


**Fig. S5 PAI of *ex vivo* tissues of breast cancer. a** Representative US and PA images of *ex vivo* tissue pieces of mammary glands containing spontaneous breast cancer in MMTV-PyVT transgenic mice. **b** Representative US and PA images of resected MDA-MB-231 tumour tissue pieces.


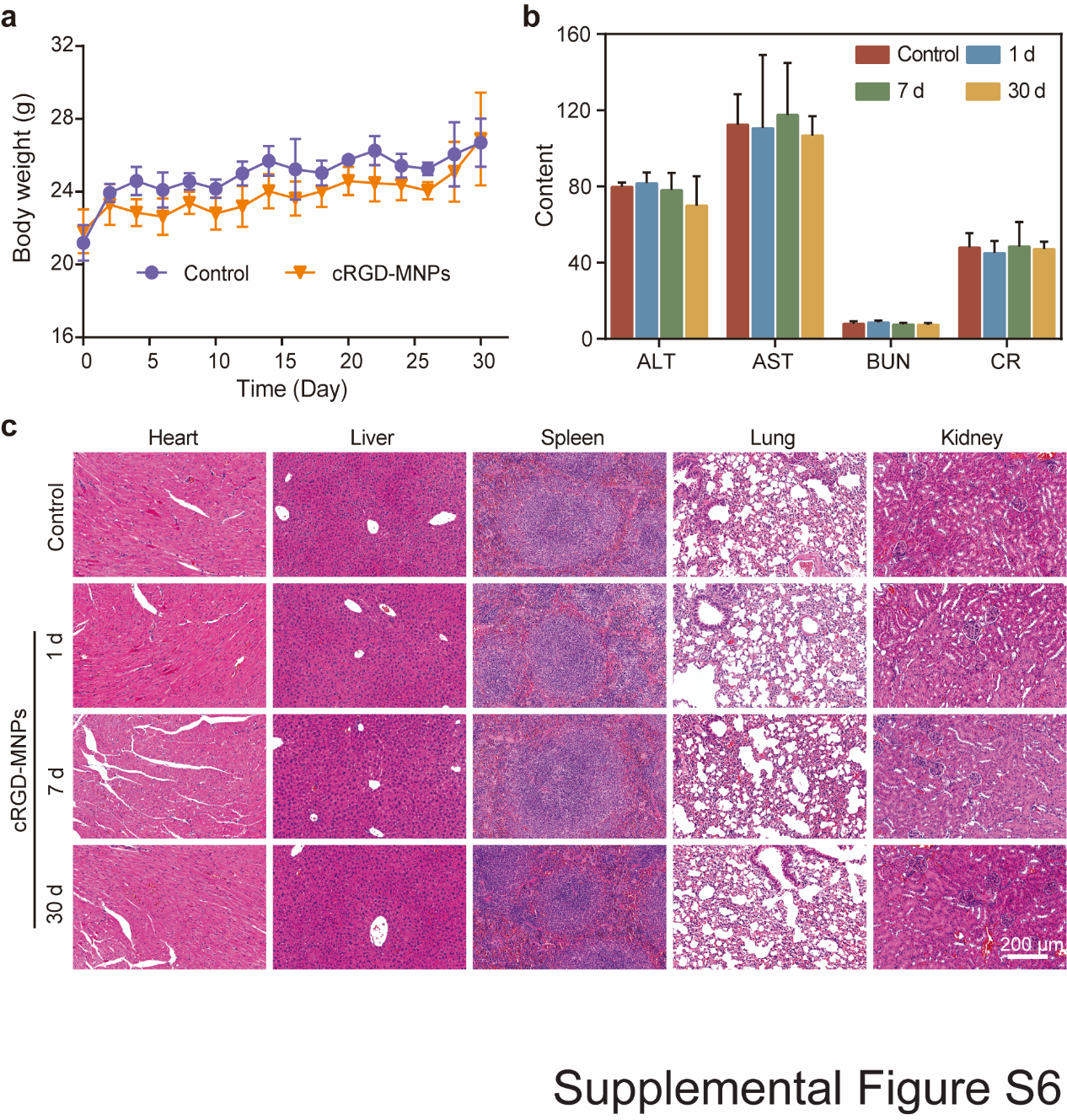


**Fig. S6 The biosafety of cRGD-MNPs. a** Body weight curves for mice treated intravenously with saline (control) or cRGD-MNPs. **b** Serum biochemistry results (liver function and renal function) for BALB/c mice at various time points (1, 7 and 30 days) after intravenous injection of cRGD-MNPs or saline (controls). The units of ALT and AST are U/L; the units of BUN and CR are mM and μM, respectively. **c** H&E staining of vital organs (heart, liver, spleen, lung and kidney) from each group. Scale bar: 200 μm. Data are presented as the mean ± SD (n = 5).
